# Mechanical and electronic properties of boron nitride nanosheets with graphene domains under strain†‡

**DOI:** 10.1039/d1ra05831b

**Published:** 2021-10-29

**Authors:** J. S. Lima, I. S. Oliveira, S. Azevedo, A. Freitas, C. G. Bezerra, L. D. Machado

**Affiliations:** Departamento de Física, Universidade Federal do Rio Grande do Norte 59078-970 Natal RN Brazil; Departamento de Física, CCEN, Universidade Federal da Paraíba Caixa Postal 5008 58051-970 João Pessoa PB Brazil; Departamento de Física, Universidade Federal do Rio Grande do Norte 59072-970 Natal RN Brazil leonardo@fisica.ufrn.br

## Abstract

Hybrid structures comprised of graphene domains embedded in larger hexagonal boron nitride (h-BN) nanosheets were first synthesized in 2013. However, the existing theoretical investigations on them have only considered relaxed structures. In this work, we use Density Functional Theory (DFT) and Molecular Dynamics (MD) simulations to investigate the mechanical and electronic properties of this type of nanosheet under strain. Our results reveal that the Young's modulus of the hybrid sheets depends only on the relative concentration of graphene and h-BN in the structure, showing little dependence on the shape of the domain or the size of the structure for a given concentration. Regarding the tensile strength, we obtained higher values using triangular graphene domains. We find that the studied systems can withstand large strain values (between 15% and 22%) before fracture, which always begins at the weaker C–B bonds located at the interface between the two materials. Concerning the electronic properties, we find that by combining composition and strain, we can produce hybrid sheets with band gaps spanning an extensive range of values (between 1.0 eV and 3.5 eV). Our results also show that the band gap depends more on the composition than on the external strain, particularly for structures with low carbon concentration. The combination of atomic-scale thickness, high ultimate strain, and adjustable band gap suggests applications of h-BN nanosheets with graphene domains in wearable electronics.

## Abstract 2

We investigate the mechanical and electronic properties of hBN nanosheets with graphene domains under strain. We find that the structures withstand large strain values and present highly adjustable band gaps, ranging from 1.0 to 3.5 eV.

## Graphical abstract

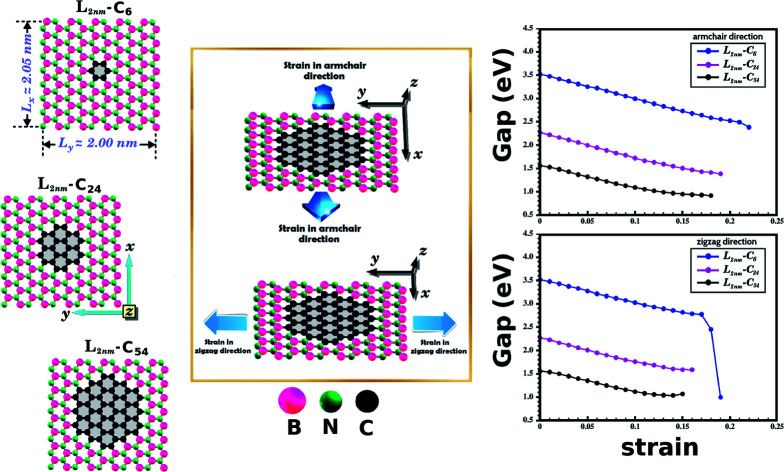

## 1. Introduction

Produced from graphite by using micromechanical cleavage technology,^1,2^ graphene is a 2D material composed of sp^2^-hybridized atoms arranged in a honeycomb-like hexagonal lattice. Various applications have been proposed for graphene in modern technologies^3,4^ due to its extraordinary properties, which include: high electron mobility at room temperature,^5^ high thermal conductivity,^6^ and broad light absorption spectrum.^7^ Moreover, graphene has motivated the search for other 2D materials arranged in a honeycomb-like hexagonal lattice. Well-known examples include hexagonal boron nitride (h-BN),^8,9^ silicene,^10^ and many transition metal dichalcogenides.^11^ In particular, h-BN, also known as white graphene, is an insulator material (energy gap >4 eV).^8,9,12^ This 2D material also presents remarkable properties, such as high thermal stability,^13^ high resistance to oxidation,^14^ and strong optical absorption in the UV region.^15,16^ Several synthesis methods have been used to achieve large area h-BN monolayers. For instance, h-BN has been grown through ultra-high vacuum chemical vapor deposition (CVD).^17,18^ We can list applications for h-BN as atomically flat substrates,^19^ as 2D dielectric materials,^21^ and in lubrication.^20^

We can certainly also include the mechanical properties of graphene and h-BN in their list of remarkable properties. Graphene is the strongest material ever synthesized, with high values of Young's modulus (≈1 TPa) and ultimate tensile strength (≈130 ± 10 GPa).^22–25^ It is also able to withstand tensile strains as large as 25%.^26^ h-BN also features high values of Young's modulus (≈0.865 TPa) and tensile strength (≈70.5 ± 5.5 GPa).^27–29^ Extensive literature exists describing the mechanical properties of graphene and h-BN and suggesting applications. For example, these structures have been used as reinforcing materials in nanocomposites^30,31^ and in highly flexible touch screens.^32^

The contrasting properties of graphene and h-BN motivated investigations on hybrid 2D materials with intermediate properties. Arrangements of C, B, and N atoms were used to propose graphene-like hexagonal sheets with h-BN nanodomains and *vice versa* (graphene/h-BN sheets).^33–39^ These structures were first synthetized by Ci *et al.* using a thermal catalytic CVD method.^40^ Since then, other synthesis methods were proposed as well.^41–43^ Regarding the mechanical, electronic, and optical properties of these hybrid structures, first-principles calculations provided values intermediate between those found in graphene and h-BN. Interestingly, these values could also be controlled by adjusting the size and geometry of the nanodomain.^33–38^ Calculations also showed that the properties of the hybrid structures depend on the graphene/h-BN interface, where the less stable C–B and C–N bonds are found.^33,37,38^ Overall, the current results indicate that graphene/h-BN sheets may be useful for applications in optoelectronic devices, as they exhibit variable band gaps (<2 eV) and an optical absorption spectrum in the visible region.

It is also possible to control the physicochemical properties of 2D materials (including graphene/h-BN hybrid sheets) through mechanical deformation.^22–25,27–29,44–50^ One way to achieve this is by applying a tensile strain, with its magnitude and direction determining whether the material undergoes bending, wrinkling, stretching, or breaking.^22,51–54^ From an experimental point of view, strain can be applied to 2D structures through various methods.^55–58^ For example, the material may be deposited in an elastic substrate, which is then elongated.^55^ As a result of the applied strain, the atomic arrangement of the system is deformed, and many properties of the material are affected by this modification. For instance, changes in thermal conductivity,^59^ electronic and optical properties,^22–25,27–29,44–48^ and chemical activity^60^ have already been reported. In turn, these changes could improve or impair the performance of a 2D material for specific applications. These developments have given rise to a new field of research called “straintronics”, which aims to control electrical properties in 2D materials through mechanical deformation.^61^

Additionally, strain exerts a significant effect on the interactions between atomic orbitals in 2D materials, and hence it greatly affects their energy band gap. For example, Fujimoto *et al.* found that a tensile strain reduces the band gap of h-BN, whereas a small compressive strain increases it.^62^ Other studies found similar results.^22,27^ In fact, each 2D material has a particular response to strain. For instance, in silicene a semimetal–metal transition was observed for biaxial strain values greater than 7.5%.^63^ For transition metal dichalcogenides, calculations and experiments investigated the effects of strain on the electronic properties, revealing a semiconductor–metal transition for large enough tensile strain values.^64,65^ And for graphene deposited on suitable substrates,^24^ strain opens the energy gap.

Strain is also often applied to materials in order to study their mechanical properties. For the hybrid graphene/h-BN sheets, Zhao *et al.*^44^ used molecular dynamics (MD) simulations to show that the fraction of h-BN contained in the structure determines its mechanical properties. In turn, this fraction depends on the geometry and/or the number of nanodomains in the sheet. They also found Young's modulus values intermediate between those of graphene and h-BN, with lower values for higher h-BN concentrations. More recently, Oliveira *et al.*^48^ combined DFT calculations and MD simulations to investigate whether the mechanical properties of hybrid sheets depend on their size. The authors found that the mechanical properties are independent of scale, so long as the graphene/h-BN concentration remained constant as the sheet size increased or decreased. Additionally, B_*x*_C_*y*_N_*z*_ hexagonal sheets with atomic arrangements that included many B–C and N–C bonds were studied with MD^49^ and DFT^50^ simulations. The reported values of stiffness and tensile strength were lower than those reported for structures composed of h-BN nanodomains embedded in graphene sheets. Regarding the effect of strain on the electronic properties of graphene/h-BN sheets, Azevedo and Kaschny determined that the band gap of this hybrid material increased with strain, from 0.08 eV to 0.5 eV.^45^ However, note that the investigated sheets were composed mainly of carbon atoms. Overall, most studies in the literature concentrate on hybrid structures composed mostly of carbon atoms.^44–50^

On the other hand, hybrid structures with high h-BN concentration have received comparatively little attention, even though reports detailing their synthesis exist.^41–43^ So far, theoretical investigations on BCN sheets with high h-BN concentration have focused on their electronic and magnetic properties.^38,39^ In the present contribution, we combine DFT calculations and MD simulations to investigate the mechanical properties as well as the effect of composition and strain on the electronic properties of h-BN sheets with graphene domains. We find that the band gaps of the investigated systems depend strongly on the graphene/h-BN concentration and moderately or weakly on the external strain (depending on the composition of the hybrid sheet). Furthermore, they can withstand strain values above 10% before permanent deformation. The combination of elasticity with a controllable band gap suggests possible applications of the investigated nanosheets as semiconductor elements on wearable electronics.

## II. Computational details and methods

**Fig. 1:**
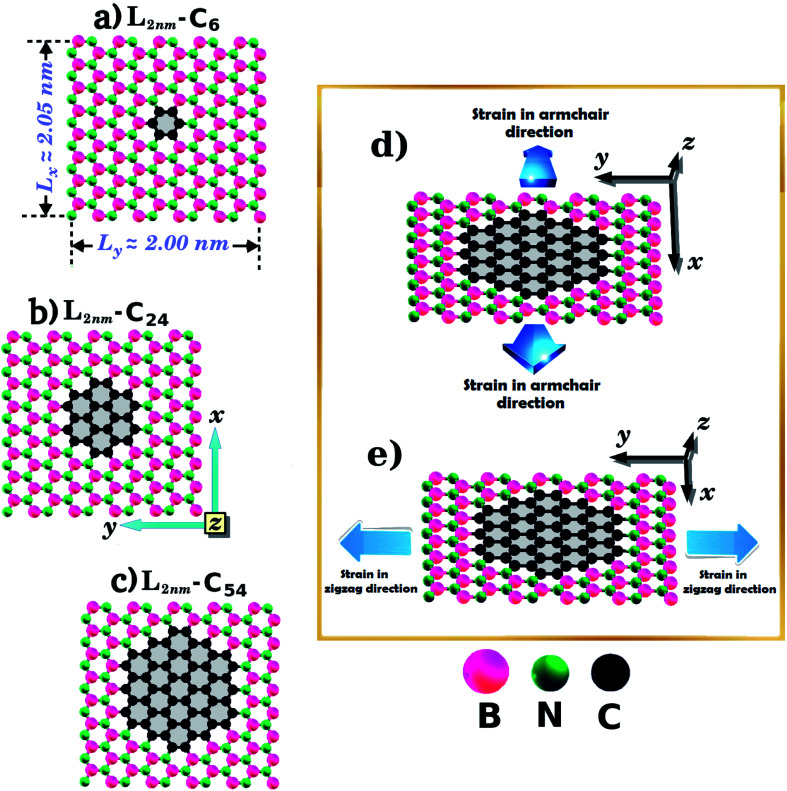
Structures investigated using both DFT and MD simulations, composed of hexagonal graphene nanodomains embedded within square h-BN monolayers, with side length *L* = 2 nm. The values of the atomic fraction of graphene (*γ*) are (a) 0.04, (b) 0.15, and (c) 0.34. The directions of the applied strain are indicated in (d) and (e).

**Fig. 2:**
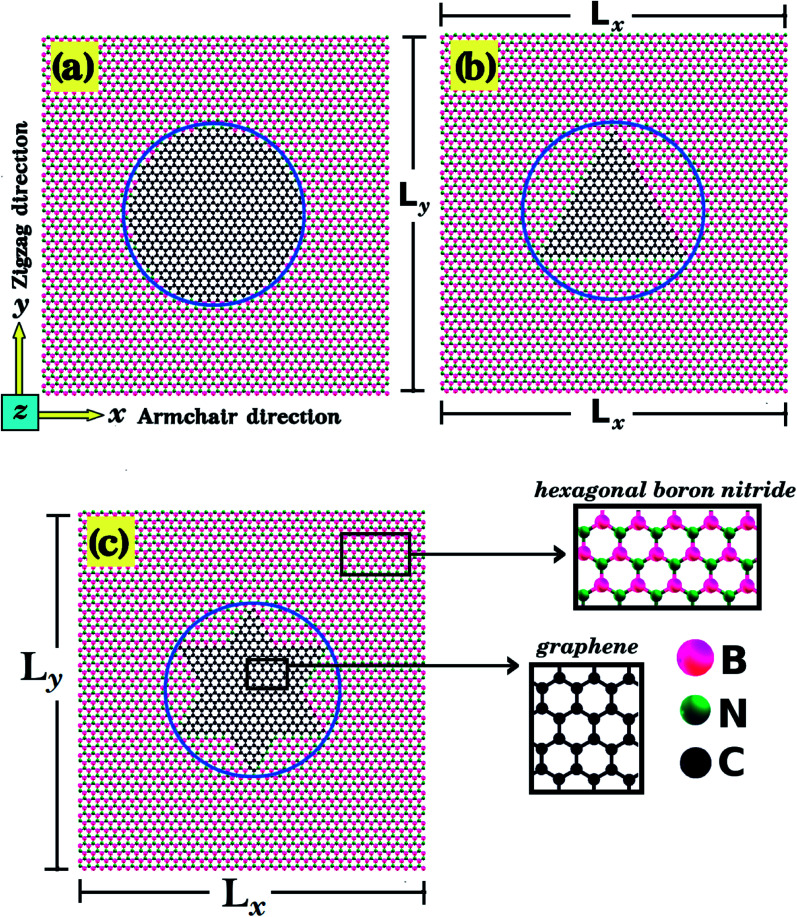
Structures investigated using MD simulations, composed of (a) circular, (b) triangular, and (c) star-shaped graphene nanodomains (inscribed within a circle of diameter *d*) embedded within square h-BN sheets (with side length *L*). We considered structures for which we kept *L* constant and varied *d* (model-I), and also structures for which we kept the ratio *d*/*L* constant and varied *L* (model-II).

In the present work, first principles calculations and MD simulations were employed to investigate the mechanical and electronic properties of h-BN nanosheets containing graphene nanodomains (h-BN/graphene sheets). Some of the studied structures are shown in Fig. 1 and 2. In our simulations, we considered square h-BN sheets (*L*_*x*_ = *L*_*y*_ = *L*), with side lengths (*L*) ranging from 2 to 50 nm (see Tables 1 and 2). In order to create the hybrid structures, we introduced graphene nanodomains in the center of the sheet by replacing B and N atoms with C atoms. Regarding the geometry of the nanodomains, for the *L* = 2 nm structure we considered hexagonal domains (see Fig. 1), whereas for the larger structures we considered circular, triangular, and star-shaped domains (see Fig. 2). We denote the number of C atoms in the graphene nanodomain by *n*_C_, which ranges from 6 to 3534 atoms. To specify a structure, we need to know both the sheet length and the graphene domain size, and so we identify each structure using the symbol *L*_*j*_–C_*x*_. For example, *L*_2nm_–C_6_ stands for the hybrid sheet illustrated in Fig. 1(a), which has 2 nm in length and a graphene nanodomain with 6 carbon atoms. Additionally, for each structure we calculated the atomic fraction of graphene (*γ*), which is given by

1 *γ* = *n*_C_/*n*_T_,

where *n*_C_ follows the previous definition and *n*_T_ is the total number of atoms. The values of *γ* for the investigated structures vary from 0.04 to 0.34, as shown in Table 1.

Calculated mechanical properties of the structures illustrated in Fig. 1(a)–(c). *L* is the side length, *γ* is the atomic fraction of graphene, *Y* is the Young's modulus, *σ* is the tensile strength, and *ε* is the ultimate strain. Results for the structures displayed in Fig. 2(a)–(c) are presented in Table 2

**Table:** 

DFT calculations	*L* (nm)	*γ*	*Y* (GPa)	*Y* (GPa)	*σ* (GPa) [*ε*]	*σ* (GPa) [*ε*]
			Armchair	Zigzag	Armchair	Zigzag
*L*_2nm_–C_6_	2	0.04	771.1	762.8	97.53 [0.22]	81.77 [0.19]
*L*_2nm_–C_24_	2	0.15	781.8	778.9	94.00 [0.19]	85.13 [0.16]
*L*_2nm_–C_54_	2	0.34	818.4	799.4	94.92 [0.19]	85.59 [0.15]

**Table:** 

MD simulations	*L* (nm)	*γ*	*Y* (GPa)	*Y* (GPa)	*σ* (GPa) [*ε*]	*σ* (GPa) [*ε*]
			Armchair	Zigzag	Armchair	Zigzag
*L*_2nm_–C_6_	2	0.04	717.5	712.2	104.5 [0.19]	89.9 [0.17]
*L*_2nm_–C_24_	2	0.15	744.5	729.6	96.5 [0.16]	85.1 [0.16]
*L*_2nm_–C_54_	2	0.34	790.3	767.6	92.9 [0.17]	80.6 [0.15]

Calculated mechanical properties of the structures illustrated in Fig. 2(a)–(c). *L* is the side length, *γ* is the atomic fraction of graphene, *Y* is the Young's modulus, *σ* is the tensile strength, and *ε* is the ultimate strain

**Table:** 

Circle	*L* (nm)	*γ*	*Y* (GPa)	*Y* (GPa)	*σ* (GPa) [*ε*]	*σ* (GPa) [*ε*]
			Armchair	Zigzag	Armchair	Zigzag
*L*_10nm_–C_3116_	10	0.189	756.5	741.0	87.92 [0.144]	89.14 [0.152]
*L*_20nm_–C_14316_	20	0.193	755.2	740.8	88.66 [0.141]	77.73 [0.125]
*L*_30nm_–C_27090_	30	0.194	753.1	739.7	86.72 [0.139]	81.23 [0.132]
*L*_40nm_–*C*_58794_	40	0.012	714.6	700.4	86.27 [0.151]	86.04 [0.160]
*L*_40nm_–*C*_56624_	40	0.049	721.6	707.8	85.56 [0.147]	75.77 [0.131]
*L*_40nm_–C_53010_	40	0.109	733.7	719.5	85.37 [0.142]	79.97 [0.136]
*L*_40nm_–C_47950_	40	0.194	752.4	736.9	87.27 [0.141]	75.67 [0.122]
*L*_40nm_–C_41444_	40	0.304	776.2	763.2	81.16 [0.124]	81.68 [0.128]
*L*_50nm_–C_74722_	50	0.195	751.1	737.7	81.14 [0.127]	81.72 [0.133]

**Table:** 

Triangle	*L* (nm)	*γ*	*Y* (GPa)	*Y* (GPa)	*σ* (GPa) [*ε*]	*σ* (GPa) [*ε*]
			Armchair	Zigzag	Armchair	Zigzag
*L*_10nm_–C_3534_	10	0.079	729.1	715.1	106.55 [0.219]	80.02 [0.141]
*L*_20nm_–C_13850_	20	0.079	727.4	714.6	106.27 [0.209]	82.95 [0.149]
*L*_30nm_–C_30948_	30	0.079	727.4	714.1	95.10 [0.179]	85.81 [0.154]
*L*_40nm_–C_59214_	40	0.005	712.7	698.5	100.11 [0.196]	81.43 [0.149]
*L*_40nm_–C_58330_	40	0.020	715.6	701.9	104.92 [0.211]	82.83 [0.152]
*L*_40nm_–C_56868_	40	0.045	720.9	706.4	101.16 [0.199]	84.53 [0.155]
*L*_40nm_–C_54759_	40	0.079	726.9	712.4	110.29 [0.225]	90.19 [0.166]
*L*_40nm_–*C*_52038_	40	0.126	736.5	720.9	97.43 [0.180]	85.43 [0.149]
*L*_50nm_–C_85318_	50	0.080	727.6	713.7	96.68 [0.181]	85.57 [0.153]

**Table:** 

Star	*L* (nm)	*γ*	*Y* (GPa)	*Y* (GPa)	*σ* (GPa) [*ε*]	*σ* (GPa) [*ε*]
			Armchair	Zigzag	Armchair	Zigzag
*L*_10nm_–C_3432_	10	0.106	735.5	721.5	93.24 [0.169]	81.01 [0.142]
*L*_20nm_–C_13454_	20	0.105	731.9	719.6	93.24 [0.140]	83.70 [0.146]
*L*_30nm_–C_30064_	30	0.105	733.3	719.4	75.87 [0.122]	79.45 [0.135]
*L*_40nm_–C_59112_	40	0.007	713.5	699.2	77.25 [0.129]	79.34 [0.142]
*L*_40nm_–C_57934_	40	0.026	716.5	702.9	83.49 [0.143]	82.44 [0.151]
*L*_40nm_–C_55984_	40	0.059	723.8	709.3	77.19 [0.127]	78.16 [0.136]
*L*_40nm_–C_53172_	40	0.107	733.0	718.6	81.86 [0.134]	78.65 [0.132]
*L*_40nm_–C_49544_	40	0.168	744.7	729.6	76.07 [0.119]	77.77 [0.127]
*L*_50nm_–C_82824_	50	0.107	733.2	719.8	75.78 [0.121]	77.63 [0.131]

All first-principles calculations are based on density functional theory (DFT),^66^ as implemented in the SIESTA code.^67,68^ Norm-conserving Troullier–Martins pseudopotentials^69^ were used, in the Kleinman–Bylander factorized form.^70^ A double-ζ polarized basis set (DZP) was used, composed of numerical atomic orbitals of finite range. The exchange–correlation energy is expressed within the generalized gradient approximation (GGA),^71^ in the form of the Perdew–Burke–Ernzerhof (PBE) functional. All geometries were fully relaxed, with residual forces smaller than 0.1 eV Å^−1^. We used a grid cutoff of 150 Ry for the grid integration, with the Brillouin zone sampled using a 10 × 10 × 1 *k*-point mesh within the Monkhorst and Pack scheme. We adopted a convergence criterion where self-consistency is achieved when the maximum difference between the output and the input of each element of the density matrix is less than 10^−4^ eV, in a self-consistent field cycle. We adopted a rectangular unit cell and imposed periodic boundary conditions. A vacuum region of 100 Å was added along the *z* direction to avoid artificial interactions between a monolayer and its periodic images.

The MD simulations were carried out using the Large-scale Atomic/Molecular Massively Parallel Simulator (LAMMPS) code.^72^ The interactions between B, C, and N atoms were described with the Tersoff potential,^73^ using the parameters adjusted by Kinaci *et al.*^74^ Our MD simulations were performed using a reasonably small timestep of 0.1 fs, and proceeded in three steps:

(1) We first evolved the system for 2 × 10^5^ steps in the *NPT* ensemble, using Nose–Hoover thermostats and barostats^75-77^ to set temperature and pressure values to 10 K and 0 Pa.

(2) We turned the thermostat off and then evolved the system for 2 × 10^5^ steps in the *NPH* ensemble, using the same barostat described above to set the pressure to 0 Pa. Note that the barostat was only applied to the planar direction that is not under strain from this step onward.

(3) In the final step we maintained the barostat configuration described above, and then elongated the system for 3 × 10^6^ steps using a strain rate of 10^−6^ fs^−1^ (for a total strain of 30%).

We used the above method to obtain stress–strain curves for graphene and h-BN. The MD results were then compared against experimental results, as detailed in the ESI.‡ Overall, we find very good agreement between our results and the experimental ones for graphene.^26^ In contrast, for h-BN, we find our results predict a lower Young's modulus and higher tensile strength when compared to the experiments.^23^ However, we note that our results are consistent with other theoretical investigations^30^

Our calculations were performed in two stages. In the first stage, we performed DFT calculations and MD simulations to determine the mechanical and electronic properties of the small hybrid h-BN/graphene sheets (*L* = 2 nm) that are shown in Fig. 1(a)–(c). In this stage we observed that both methods yielded similar stress–strain curves. So, in the second stage, we only performed MD simulations to investigate the mechanical properties of larger hybrid sheets (10 ≤ *L* ≤ 50 nm), which are shown in Fig. 2. In turn, we divided the calculations with the larger structures in two models. In model-I, the side length *L* is constant and the graphene/BN concentration is variable, while in model-II the side length *L* is variable and the graphene/BN concentration is constant. In both models, we considered different geometries (circular, triangular, and star-shaped). In order to determine the mechanical properties of these structures, we applied a tensile strain along one direction and then calculated the resulting tensile stress to obtain a stress–strain curve. Next, we calculated the slope of the linear region to determine the Young's modulus (*Y*). The tensile strength (*σ*) and the ultimate strain (*ε*) were taken at the point where the stress reaches its maximum value. The calculated values of *Y*, *σ*, and *ε* are presented in Tables 1 and 2.

One final remark is that the methodology used to obtain the stress is different for DFT and MD simulations. For the DFT approach, we first increased one side of the simulation box by 1% and then relaxed both the other side and the atomic positions until the calculation converged. After that, we increased the simulation box by 1% again and repeated the process. For the MD simulations, we increased one direction of the simulation box continuously at a fixed strain rate, and used a barostat to keep the other direction relaxed. Meanwhile, we allowed all atoms to freely evolve. Strain was applied along the armchair (*x*) and zigzag (*y*) directions, as illustrated in Fig. 1(d), (e) and 2(a).

## III. Results and discussion

**Fig. 3:**
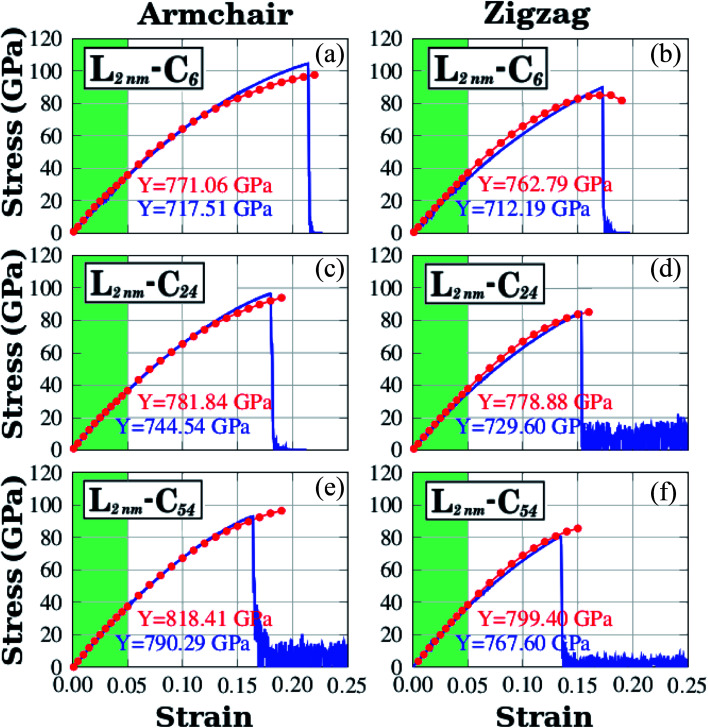
Stress–strain curves obtained through DFT and MD simulations for the structures shown in Fig. 1. In plots (a), (b), and (c) strain is applied to the armchair direction; while in plots (d), (e), and (f) strain is applied to the zigzag direction. For DFT results, the red solid circles indicate data points and the line is only a guide to the eye. For MD results, the number of data points is large, and a blue line is used to connect adjacent points. The green region corresponds to the elastic region.

**Fig. 4:**
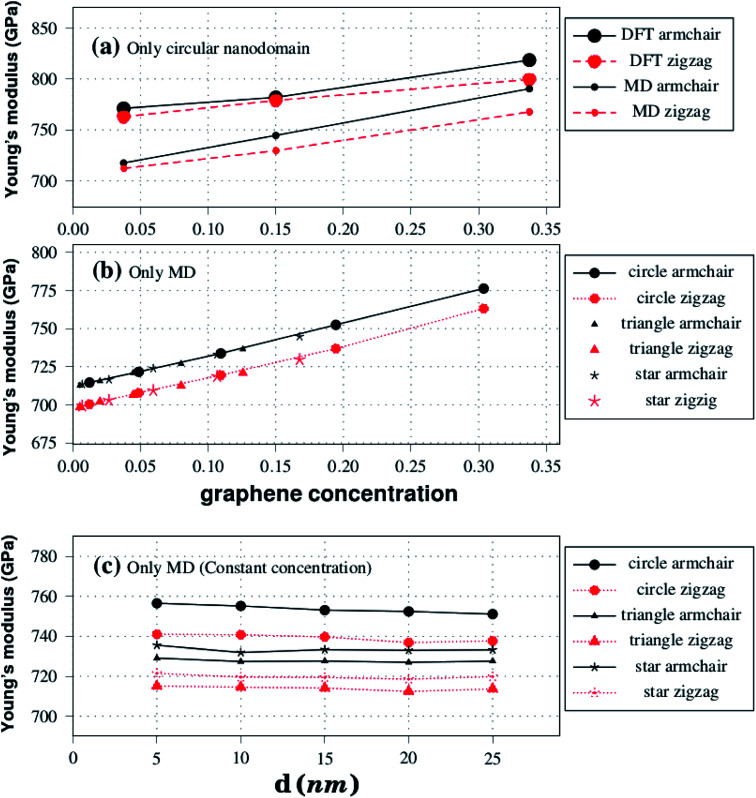
Plot (a) shows Young's modulus results against graphene concentration for the structures displayed in Fig. 1. Plots (b) and (c) show Young's modulus results against graphene concentration and side length, respectively, for the structures shown in Fig. 2.

We have used DFT calculations and MD simulations to analyze the mechanical properties of the structures displayed in Fig. 1(a)–(c) (*L*_2nm_–C_6_, *L*_2nm_–C_24_, and *L*_2nm_–C_54_). For each structure, we obtained stress–strain curves for both the armchair and zigzag directions which are shown in Fig. 3(a)–(f). In Fig. 3 we highlight in green the linear elastic region, which occurs for strain values between 0 and 0.05 (or 5%). Observe the good agreement between the DFT and MD results in this region. This agreement is likely related to the fact that the parameters used for the MD simulations were parameterized to reproduce the results of DFT calculations.^74^ As a consequence, the difference between Young's modulus values obtained through DFT and MD is only ∼10% (see Fig. 4(a)). We found a non-linear relationship between stress and strain for higher strain values, even though no atomic rearrangement occurred, which would indicate permanent deformation. Also, notice that the agreement between the DFT and MD results is not as good in this non-linear region, although the disparity is still reasonably small. We attribute these differences to distinct DFT and MD methodologies (for instance, we use non-zero temperatures in the MD simulations). As the strain continues to increase, eventually the material fractures for strain values between 15% and 22%. Both DFT and MD results predict that the maximum tensile strain is higher along the armchair direction than along the zigzag direction. The stress–strain curves obtained here are similar to other curves reported in the literature.^44–50^

**Fig. 5:**
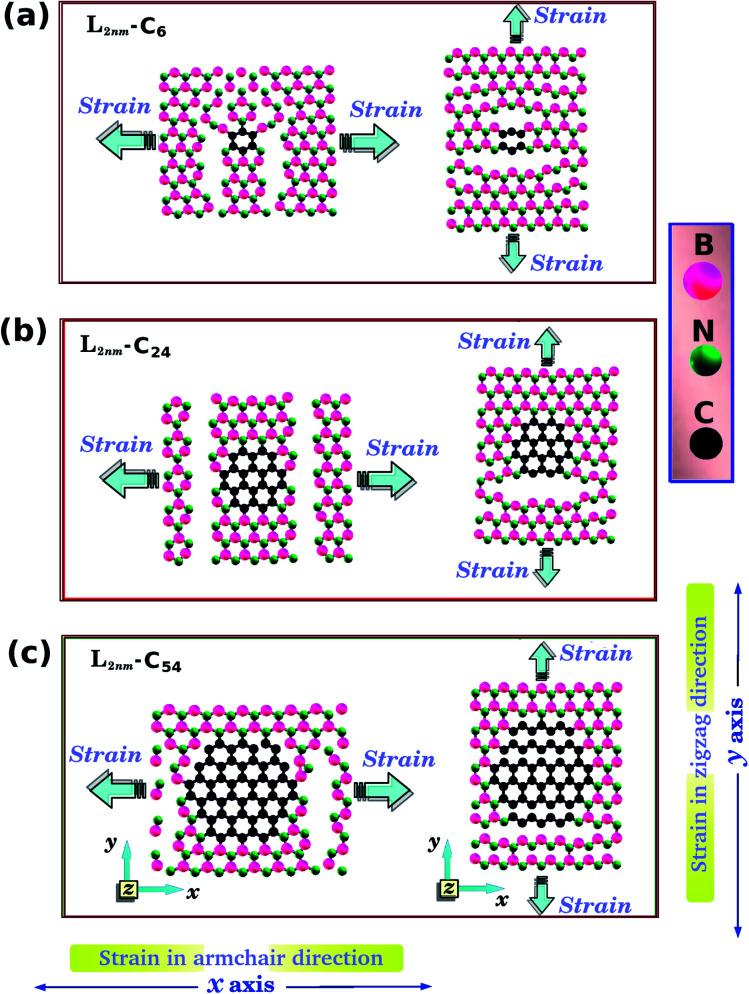
Snapshots from DFT calculations displaying the fracture patterns obtained for *L*_2nm_–C_6_, *L*_2nm_–C_24_, and *L*_2nm_–C_54_. The arrows in this figure indicate the directions in which we applied strain.

Let us discuss in more detail the DFT predictions for the mechanical properties of the h-BN nanosheets containing graphene domains. The Young's modulus (*Y*) and tensile strength (*σ*) values obtained for *L*_2nm_–C_6_, *L*_2nm_–C_24_, and *L*_2nm_–C_54_ are summarized in Table 1. Comparing the results obtained for the zigzag and armchair directions, we find that the mechanical properties of the hybrid sheets are weakly anisotropic, with differences of ∼2% for Y and ∼10% for *σ*. Note that the same behavior was observed for h-BN, graphene, and other h-BN/graphene sheets, since this weak anisotropy is related to the hexagonal structure of the unit cell.^22–25,27–29,44–50^ Our results also show that the Young's modulus values increase with increasing concentrations of graphene, regardless of the direction of applied strain. In contrast, we find no conclusive relation between the tensile strength and ultimate strain with the number of C atoms. These results are in agreement with others previously reported for graphene/BN sheets, which found higher Young's modulus for higher concentrations of graphene.^44,45,48^ After the stress reaches its maximum value, for strain values of 21% for the armchair and 15% for the zigzag direction, the h-BN/graphene sheets fracture (see Fig. 5). Regarding the obtained fracture patterns, we find similar results for the zigzag and armchair directions. Bonds initially break in the vicinity of the nanodomain, and then the fracture propagates along a direction that is perpendicular to the applied strain. In this process, C–B, C–N, and B–N bonds are broken, whereas the graphene domains remain intact. After fracture occurs, the DFT calculations no longer converge.

Let us now discuss the mechanical properties of the larger hybrid sheets (10 ≤ *L* ≤ 50 nm), which are shown in Fig. 2. Due to their large size, only MD simulations were used to investigate their mechanical properties. Note, however, that the comparison between DFT and MD results for the smaller structures indicates that the MD results are reliable, particularly in the linear elastic region. We consider two models (model-I and model-II) for the larger graphene/BN sheets. In model-I we keep the size of the hybrid sheet constant (*L* = 40 nm) while we vary the domain size (*γ* ranges from 0.07 to 0.304, see Table 2). The goal of model-I is to investigate the dependence of the mechanical properties on composition. In model-II we keep the fraction of graphene constant (*γ* = 0.19) while we vary the sheet size (10 ≤ *L* ≤ 50 nm). The goal of model-II is to investigate possible size effects (the ratio of hybrid C–B and C–N bonds to the total number of bonds decreases as the sheet size increases). We also consider whether the shape of the domain affects the mechanical properties for both model-I and model-II. The mechanical properties of the larger sheets are summarized in Table 2. These results were extracted from various stress–strain curves, which can be found in Fig. S2 of the ESI.‡ Our results indicate that the linear region for the larger structures extends from 0 to 3%.

Regarding model-I, we find that the Young's modulus increases as the concentration of graphene increases, whereas we find no clear relationship between tensile strength and composition. Young's modulus results for model-I are summarized in Fig. 4(b). Observe that *Y* increases linearly with the concentration of graphene for both armchair and zigzag directions. Comparing results for both directions, we again notice that the Young's modulus is slightly higher for the armchair direction. Regarding the domain shape, we find that it has no noticeable effect on Young's modulus values, other than the fact that different shapes have distinct concentrations of graphene. This conclusion is supported by the data presented in Fig. 4(b). Notice that all Young's modulus results obtained fall within the same line, irrespective of the domain shape. We discuss possible reasons for the lack of relationship between Young's modulus values and domain shape in the ESI.‡

Moving on to the tensile strength results for model-I, they are summarized in Table 2. Observe that *σ* values vary as the domain size increases, but with no clear trend, sometimes increasing and sometimes decreasing as the graphene concentration increases. This occurs because the tensile strength of the hybrid sheets is limited by the weaker B–C and C–N bonds (the fracture process is described ahead). Finally, note that, unlike the Young's modulus, the tensile strength has a weak dependence on the domain shape, being overall higher for triangular shapes when comparing structures with similar *γ* values. Furthermore, while we did not study the effect of the shape on the electronic properties, previous investigations on this topic found that shape affects these properties.^33^

Regarding model-II, we find that the mechanical properties of the BN nanosheets with graphene domains do not depend on the sheet size, as long as the graphene concentration remains constant as the sheet size varies. Young's modulus results for model-II are presented in Fig. 4(c). For constant *γ*, notice that the Young's modulus remains constant as the size of the nanosheet increases, for all pulling directions and domain shapes. Inspection of Fig. 4(c) also reveals that the Young's modulus is yet again slightly higher along the armchair direction. Also, notice that *Y* is higher for circular and lower for triangular domains. However, it is important to remark that although *γ* is constant for structures with the same domain shape, it does vary for structures with different domain shapes. For model-II, we used *γ* values of 0.19, 0.08, and 0.11 for circular, triangular, and star-shaped domains, respectively. Hence, model-II structures with circular domains are stiffer simply because they contain more graphene. With regard to the tensile strength, we find no clear relationship between this quantity and the nanosheet size in the results presented in Table 2. Finally, the tensile strength appears to be higher for structures with triangular domains, particularly when strain is applied along the armchair direction.

We have also considered systems with lower symmetry than those discussed so far. We used MD simulations to study h-BN monolayers with (i) asymmetrical graphene domains and (ii) rotated triangular graphene domains. The results obtained for structures with lower symmetry are presented and discussed in the ESI.‡ In general, we continue to find Young's modulus values intermediate between h-BN and graphene. However, we found a higher degree of anisotropy for the mechanical properties of the asymmetrical structure. We also found that the orientation of the triangular domain does not affect Young's modulus results but does affect tensile strength and ultimate strain values.

**Fig. 6:**
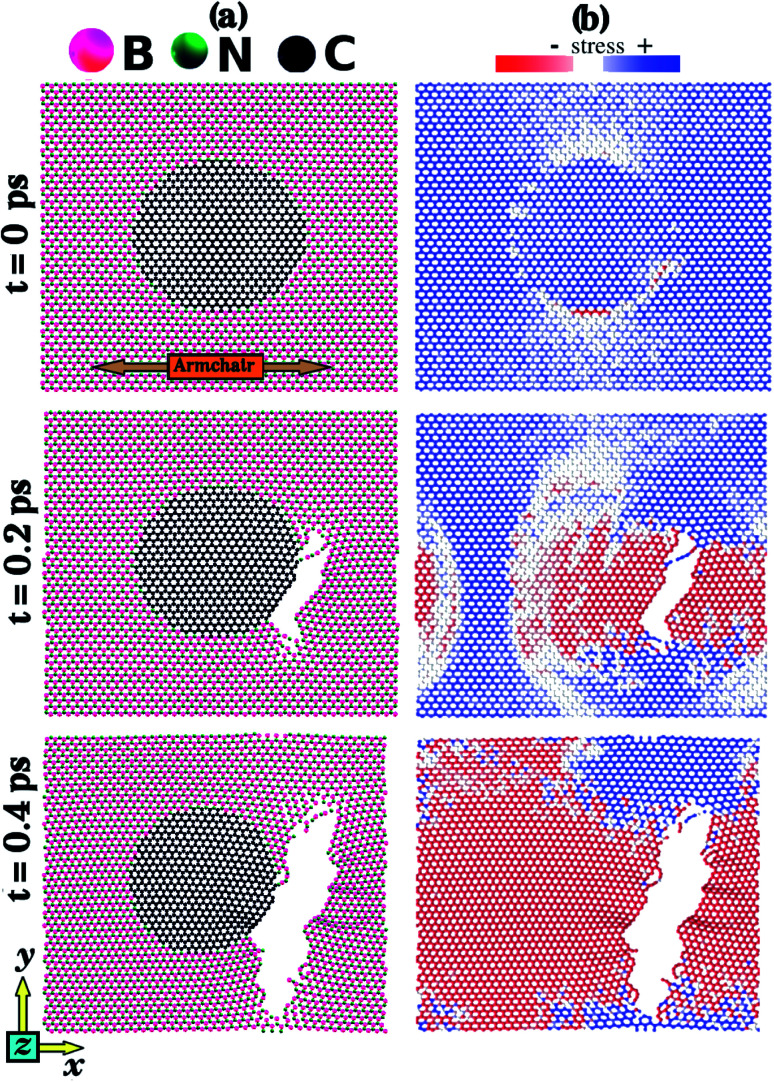
Fracture results, for strain applied along the armchair direction for the hybrid sheet with *L*_*x*_ = *L*_*y*_ = 10 nm and a circular graphene domain (*d* = 5 nm). (a) Snapshots from MD simulations detailing the time evolution of the monolayer fracture. (b) Corresponding stress distribution for the structures presented in (a).

Fig. 6 shows the stress and fracture patterns obtained in an MD simulation where the strain was applied along the armchair direction. Fig. 6(a) shows atomic configurations while Fig. 6(b) shows the corresponding stress distributions. Similar results for the zigzag direction are presented in Fig. S4 of the ESI.‡ Since the overall behavior is the same for both directions, we only detail armchair results. Before fracture the stress is higher (blue) in the middle region of the hybrid sheet, whereas the stress is lower (white) in the regions above and below the graphene domain. To understand this result, notice that if we think of the sheet as composed of multiple thin segments spanning its length along the *x*-direction, we find that each thin segment is under the same strain (as the entire sheet is under the same strain). Hence, as the regions above and below the graphene domain are composed entirely of the less stiff h-BN, lower stress values produce the same strain. Consequently, fracture starts in the middle region, where stress is higher on average (*t* = 0 ps). However, note that strain is not particularly high at the graphene–BN interface, where the fracture always begins. This region is where we find the weaker hybrid bonds, and the C–B bonds were always the first to break. To understand this result, we note a previous DFT study that calculated first-neighbor bond energies for C–C, B–N, C–N, and C–B bonds, for hybrid B_*x*_C_*y*_N_*z*_ structures.^78^ The authors found that the C–B bond had the lowest bond energy. Fracture then propagates perpendicular to the applied strain (*t* = 0.2 ps). This result is in agreement with the previously discussed DFT results. As the process continues, stress decreases (*t* = 0.4 ps).

**Fig. 7:**
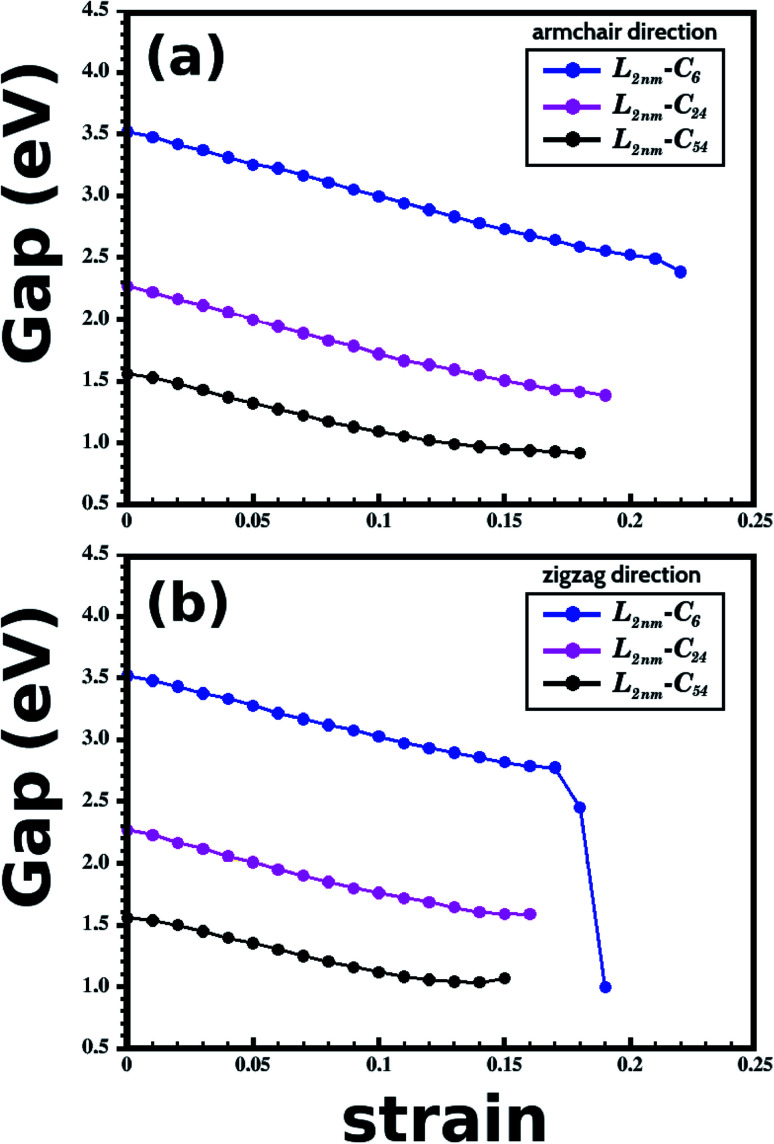
Results of energy band gap *versus* strain for the structures presented in Fig. 1. (a and b) Show results for the case where the strain was applied to the armchair and zigzag directions, respectively.

**Fig. 8:**
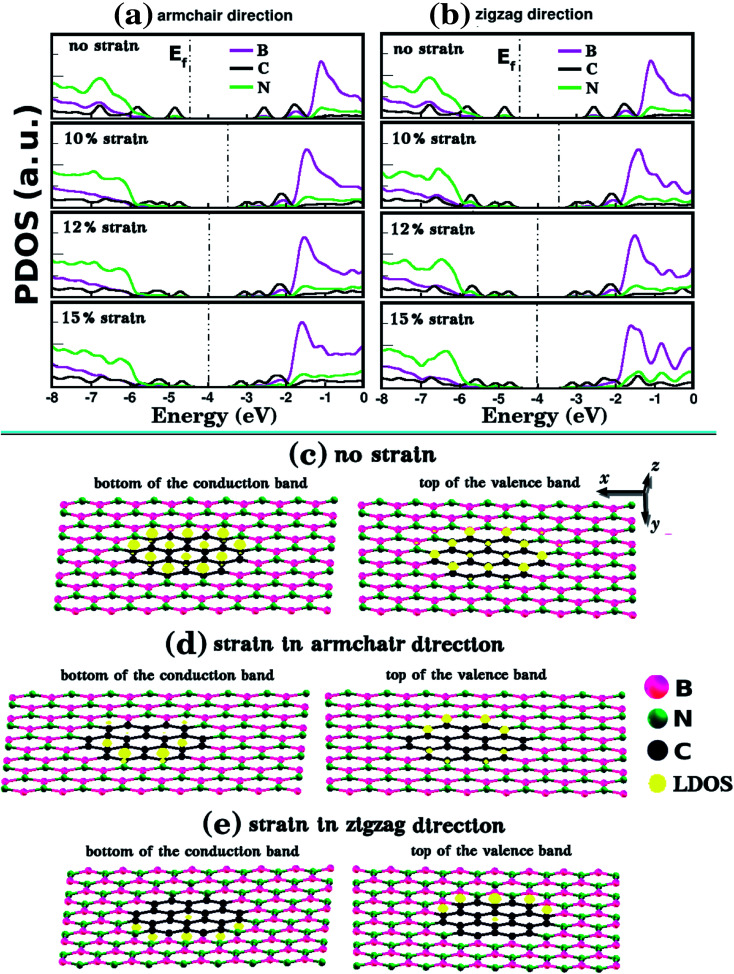
Plots (a) and (b) show the projected density of states (PDOS) for structure *L*_2nm_–C_24_ for different strain values. In (a), the strain was applied to the armchair direction and in (b) to the zigzag direction. The dotted vertical line indicates the Fermi energy *E*_f_. (c) Shows the local density of states (LDOS) associated with the bottom of the conduction band and the top of the valence band for the *L*_2nm_–C_24_ monolayer without strain. (d and e) Show the LDOS of *L*_2nm_–C_24_ for strains applied to the armchair and zigzag directions, respectively.

Let us now discuss the DFT predictions for the electronic properties of the structures shown in Fig. 1(a)–(c). Fig. 7(a) and (b) present the energy band gap *E*_g_ against the strain for the armchair and zigzag directions. These values were calculated from the electronic band structures with 100 *k*-points along the *Γ*–*X*–*Z* direction. For zero strain, each structure has the same value of *E*_g_ in both directions, as expected. The gap values for *L*_2nm_–C_6_, *L*_2nm_–C_24_, and *L*_2nm_–C_54_ are 3.52 eV, 2.27 eV, and 1.56 eV, respectively. Comparing the smaller graphene domain with the others, we find a decline in the band gap of 35.5% for *L*_2nm_–C_24_ and 55.7% for *L*_2nm_–C_54_. This energy gap reduction for increased concentrations of graphene is in agreement with the results found by Manna *et al.*^33^ and Azevedo *et al.*^37^Fig. 8(a) shows the projected density of states (PDOS) for selected h-BN nanosheets containing graphene nanodomains. We can infer from the PDOS that the electronic states close to the Fermi energy *E*_f_ are in general associated with C atoms. On the other hand, B and N atoms contribute to the electronic states in the conduction and valence bands, respectively. In agreement with the PDOS, the localized density of states (LDOS) results presented in Fig. 8(b) reveal that the bottom of the conduction band and the top of the valence band are associated with p_*z*_ orbitals from C atoms. These results are common features of hybrid nanostructures with stoichiometry B_*x*_C_*y*_N_*z*_.^12,36,37^ Finally, note that we did not calculate the band gap of the larger structures presented in Fig. 2 because they were only investigated using classical molecular dynamics methods, which cannot be used to calculate electronic properties.

In general, we observe that the energy gap decreases gradually as the applied strain increases, except in the case of *L*_2nm_–C_6_ for strain values >16%, where we observed a sharp gap decline. The following discussion on the band gap variation with strain is concentrated on results for values below 15%, since fracture becomes an issue for some structures for larger strain values. In general, the energy gap decreases slightly more when the strain is applied to the armchair direction. Furthermore, we observe that the band gap of the considered structures varies weakly or moderately with deformation. These results can be seen more easily in Table 3, which provides band gap values and percentage variations for selected strain values. Examining these results, we notice that the gap values become more sensitive to the external strain as we increase the fraction of graphene in the hybrid sheet. The relatively small gap variation for structures with a high concentration of h-BN is expected, since the band gap of this material presents small variation with the strain for a uniaxial strain below 15%.^79^ In contrast, the band gap of other 2D materials can vary by more than 50% for uniaxial strain values around 10%. For example, this is the case for many transition metal dichalcogenides^80^ (the smallest variation was observed for MoTe_2_, for which the band gap decreased around 35% for 10% strain). Finally, when comparing the effects of composition and strain, we find that the former has a more substantial influence on the band gap (except for the case of *L*_2nm_–C_6_ deformed along the zigzag direction). We can also combine both effects to decrease the band gap from 3.5 eV to values below 1.0 eV.

**Table:** Electronic band gap (*E*_g_) of the structures illustrated in Fig. 2(a)–(c) for selected strain values. The numbers in parenthesis correspond to the percentage variation relative to the initial value

DFT calculations	Armchair 5%	Armchair 10%	Zigzag 5%	Zigzag 10%
	*E*_g_ (eV)	*E*_g_ (eV)	*E*_g_ (eV)	*E*_g_ (eV)
*L*_2nm_–C_6_	3.25 (7.67%)	3.00 (14.8%)	3.28 (6.82%)	3.02 (14.2%)
*L*_2nm_–C_24_	2.00 (11.9%)	1.72 (24.2%)	2.01 (11.5%)	1.76 (22.5%)
*L*_2nm_–C_54_	1.32 (15.4%)	1.09 (30.1%)	1.35 (13.5%)	1.12 (28.2%)

An important note is that the DFT method underestimates the band gap of semiconductors,^81^ and consequently, the numbers presented here are underestimated. However, we remark that other articles calculated band gaps of strained structures using different methods and found that, while the band gap values depend on the method employed, the general trends do not.^79,82^ The size of the structures considered here in the DFT calculations (160 atoms) hampered the use of more accurate methods.

Altogether, the calculated properties of the h-BN nanosheets with graphene domains indicate possible uses in wearable electronics. For such applications, a material needs to (i) be stretchable, (ii) be flexible, (iii) operate under large deformation.^83^ Regarding requirement (i), our calculations show that the investigated monolayers can be extended by at least 10% before undergoing permanent deformation. Concerning condition (ii), although the hybrid sheets are very stiff, they are also very thin, requiring small forces to deform (for example, flexible silicon devices have been produced by reducing their thickness^84^). Finally, regarding requirement (iii), our results show that the considered structures remain semiconductors over a wide range of strain values. They also reveal that it is possible to control the band gap of the considered monolayers by changing their composition. Semiconductors have been used in wearable electronics as photodetectors,^83^ as transistors,^85^ and as components in sensors.^86^ Therefore, h-BN nanosheets with graphene domains may play an important role in wearable electronics.

## IV. Conclusions

In summary, we combined DFT and MD simulations to investigate the mechanical and electronic properties of h-BN nanosheets with graphene domains under strain. Regarding the mechanical properties, we found good agreement between the DFT calculations and MD simulations – the former was used to study smaller systems and the latter to study both smaller and larger structures. We found that Young's modulus values depend only on the relative concentration of graphene and h-BN. It does not depend on either the shape of the domain or the size of the structure (provided the h-BN and graphene domain are increased/decreased proportionately). Our results also revealed that the tensile strength is slightly higher for triangular graphene domains, particularly when applying strain to the armchair direction. In the case of DFT calculations, the system fractures for strain values between 15% and 22% at the junction between h-BN and graphene. In the case of MD calculations, the ultimate strain was slightly lower, but fracture also starts in the same region, even though stress is not higher at the interface. Fracture begins at the junction between h-BN and graphene because it is the location of the weaker C–B bonds.

Regarding the electronic properties, we found that the band gap depends more on the composition of the hybrid sheets than on the external strain. Our results also show that the band gap of structures with more carbon atoms is more sensitive to the applied strain. However, the variation of the band gap with the external strain is still relatively small compared to other 2D materials. Furthermore, when composition and strain are combined to modify the band gap, we can obtain a broad range of values (from 1.0 eV to 3.5 eV). The proposed hybrid monolayers combine elasticity (the nanosheets can withstand considerable stress before plastic deformation), atomic-scale thickness (allowing the monolayers to bend and deform easily), and adjustable band gap (allowing the customization of the material for different applications). Those properties indicate that the h-BN nanosheets with graphene domains could be promising materials for applications in wearable electronics.

## Conflicts of interest

There are no conflicts to declare.

## Supplementary Material

RA-011-D1RA05831B-s001
