# Supplementary material for: Mechanical and electronic properties of boron nitride nanosheets with graphene domains under strain
Source: RSC Adv. 2021 Oct 29;11(56):35127–40. doi: 10.1039/d1ra05831b (PMC9042849; doi:10.1039/d1ra05831b)
Supplement: RA-011-D1RA05831B-s001 [file RA-011-D1RA05831B-s001.pdf]

Electronic Supplementary Material (ESI) for RSC Advances.
This journal is © The Royal Society of Chemistry 2021

Electronic Supplementary Information: Mechanical and electronic properties of boron nitride nanosheets with graphene domains under strain

J. S. Lima,[1] I. S. Oliveira,[2] A. Freitas,[1] C. G. Bezerra,[1] S. Azevedo,[2] and L. D. Machado[1,*]

[1]*Departamento de Física, Universidade Federal do Rio Grande do Norte, 59072-970, Natal, RN, Brazil.*

[2]*Departamento de Física, CCEN, Universidade Federal da Paraíba, Caixa Postal 5008, 58051-970, João Pessoa, PB, Brazil.*

(Dated: September 25, 2021)

CONTENTS

Molecular dynamics results: stress-strain curves for graphene and h-BN and comparison to experimental results 3

Molecular dynamics results: stress-strain curves 5

Possible explanation for the lack of relationship between the Young's modulus and the domain shape. 6

Molecular dynamics results: structures with lower symmetry 7

Molecular dynamics results: fracture for the zigzag direction 9

References 10

MOLECULAR DYNAMICS RESULTS: STRESS-STRAIN CURVES FOR GRAPHENE AND H-BN AND COMPARISON TO EXPERIMENTAL RESULTS

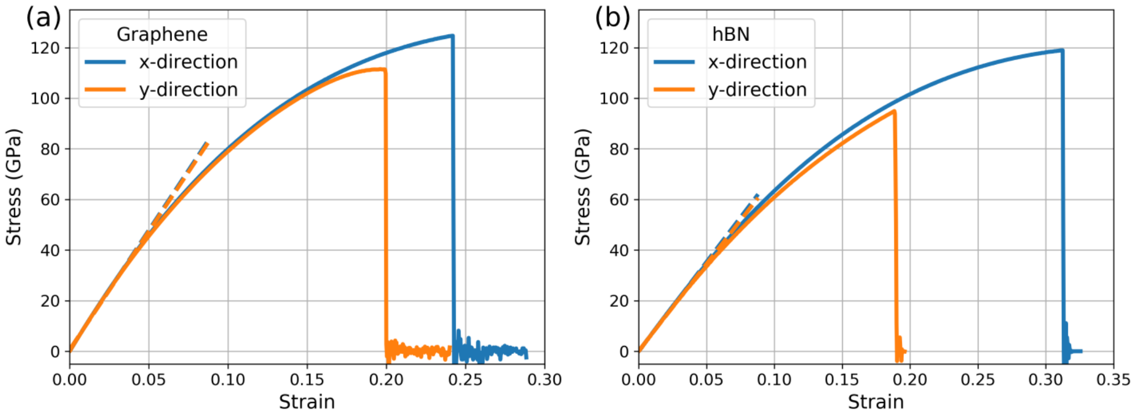

FIG. S1. Stress–strain curves obtained using MD simulations for (a) graphene and (b) h-BN. The blue (orange) curves correspond to results for the armchair (zigzag) directions.

This section compares simulation and experimental results for the mechanical properties. Towards this end, we used MD simulations to obtain stress-strain curves for graphene and h-BN and present the results in Fig. S1. The obtained mechanical properties are summarized in Table II, which also includes experimental values. Regarding the experimental results, note that the presented values are not the result of tensile tests. Instead, the data is the outcome of nanoindentation experiments interpreted using analytical models. One consequence is that deformation is not uniaxial, and results cannot be categorized as either armchair or zigzag.

Let us first compare MD and experimental results for graphene. In this case, we find quite a good agreement between the two data sets for all mechanical properties. On the other hand, the agreement is not as good for h-BN. We find the simulations underestimate the Young's modulus and overestimate the tensile strength, particularly for the armchair direction. However, we remark that the hybrid nanosheets fracture at tensile strengths lower than those predicted here for h-BN due to the weaker B-C bonds. Finally, we note that the values obtained here are closer to other simulation results. For instance, the values provided in a recent review article [3] were:

1. Young's modulus: 797 GPa (assuming a monolayer thickness of 0.34 nm)

2. tensile strength: up to 99.0/85.4 GPa in the x-direction/y-direction (assuming a monolayer thickness of 0.34 nm)

3. ultimate strain: 0.29/0.18 in the x-direction/y-direction

TABLE I. Comparison between MD and experimental results for the mechanical properties of graphene and h-BN. Y is the Young's modulus, σ is the tensile strength, and ϵ is the ultimate strain.

		**Armchair**	
**MD Simulations**	**Y** (GPa)	σ (GPa)	ϵ
**Graphene**	946	125	0.24
**h-BN**	704	119	0.31
		**Zigzag**	
**MD Simulations**	**Y** (GPa)	σ (GPa)	ϵ
**Graphene**	936	112	0.20
**h-BN**	687	95	0.19
**Experimental results**	**Y** (GPa)	σ (GPa)	ϵ
**Graphene (ref. [1])**	1000	130	0.25
**h-BN (ref. [2])**	865	70.5	0.17

MOLECULAR DYNAMICS RESULTS: STRESS-STRAIN CURVES

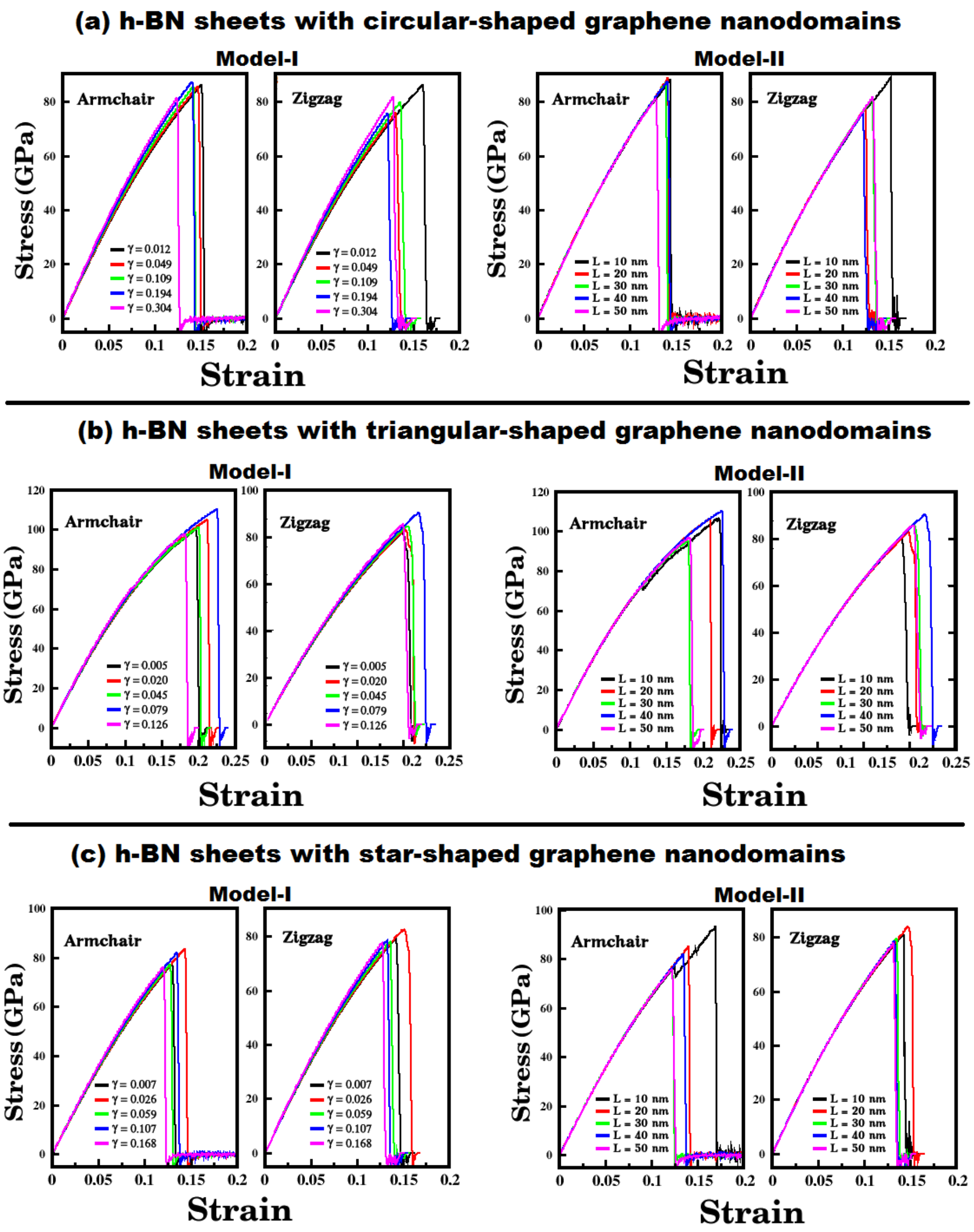

FIG. S2. Stress–strain curves obtained using MD simulations. Plots (a), (b), and (c) show the results for h-BN sheets with circular, triangular, and star-shaped graphene nanodomains.

POSSIBLE EXPLANATION FOR THE LACK OF RELATIONSHIP BETWEEN THE YOUNG'S MODULUS AND THE DOMAIN SHAPE.

This section discusses qualitatively why we find no dependence between Young's modulus values and domain shape. We believe this is related to both the elastic regime and the low number of C-B and C-N bonds. Regarding the first factor, our results indicate that each bond acts as a restorative spring independently of other bonds in this regime. In this case, the stress needed to produce a particular strain would normally depend on the proportion of C-C, B-N, C-B, and C-N bonds. However, since the number of C-B and C-N bonds is small, their contribution is minimal. If each bond requires a specific force to achieve a particular strain, a larger fraction of stiffer bonds (C-C) would mean higher total force and stress values. Consequently, structures with more C-C bonds should have higher Young's modulus values. In contrast, an increased fraction of less stiff bonds (B-N) would mean lower total force and stress values and, therefore, structures with lower Young's modulus values.

MOLECULAR DYNAMICS RESULTS: STRUCTURES WITH LOWER SYMMETRY

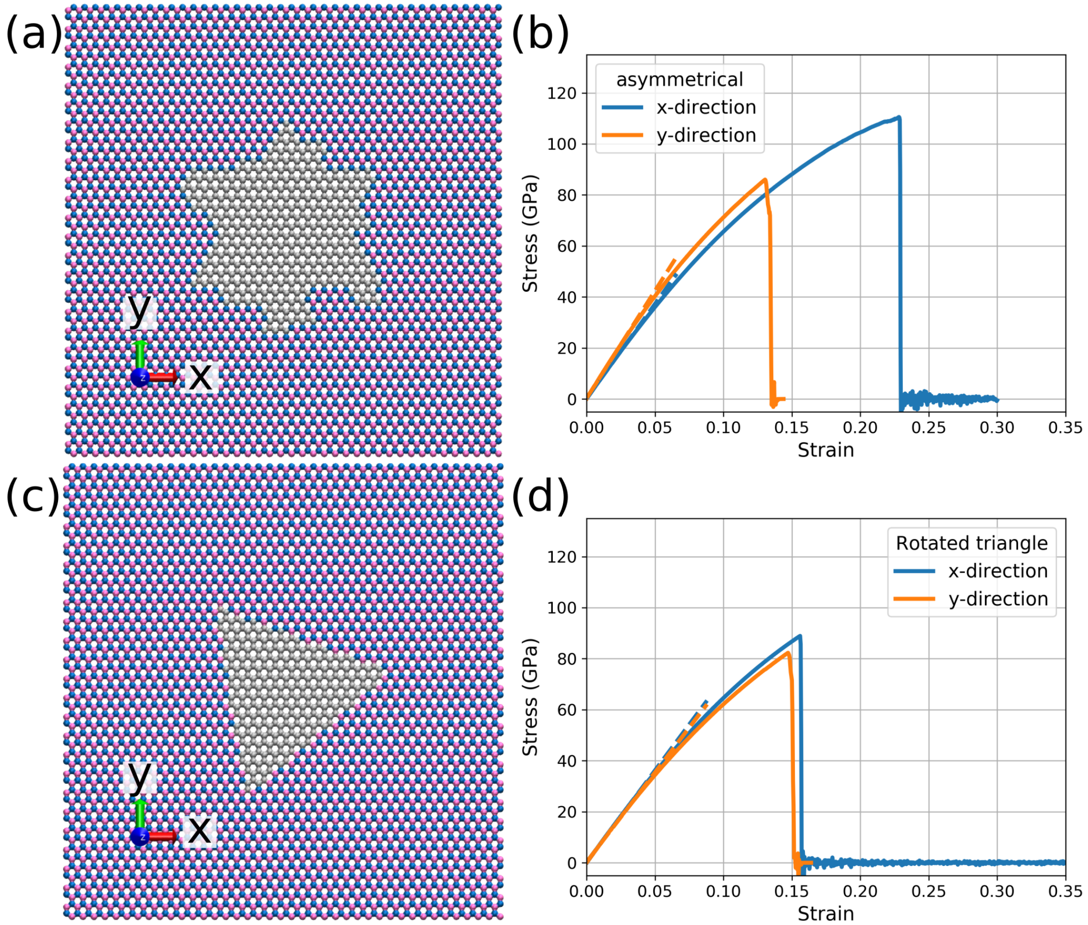

FIG. S3. (a) and (c) present hybrid BNC structures with lower symmetry. (b) and (d) present stress-strain curves obtained for these structures using MD simulations, while table II summarizes their mechanical properties. We find once again Young's modulus values between h-BN and graphene. In comparison to calculations performed for systems with high symmetry, we observe a higher degree of anisotropy in the mechanical properties of the asymmetrical structure. For the triangular structure, we find the Young's modulus results are similar to those obtained for a structure with equal size and composition (L_{10nm}-C_{3534}). However, the tensile strength and strain values are rather different, indicating that the domain orientation affects the results for these mechanical properties.

TABLE II. Molecular Dynamics results for the mechanical properties of the lower symmetry structures. Y is the Young's modulus, σ is the tensile strength, and ϵ is the ultimate strain.

	**Armchair**			**Zigzag**		
**MD Simulations**	**Y** (GPa)	σ (GPa)	ϵ	**Y** (GPa)	σ (GPa)	ϵ
**asymmetrical structure**	743	110	0.23	842	86	0.13
**rotated triangle**	720	88.9	0.16	702	82.3	0.15

MOLECULAR DYNAMICS RESULTS: FRACTURE FOR THE ZIGZAG DIRECTION

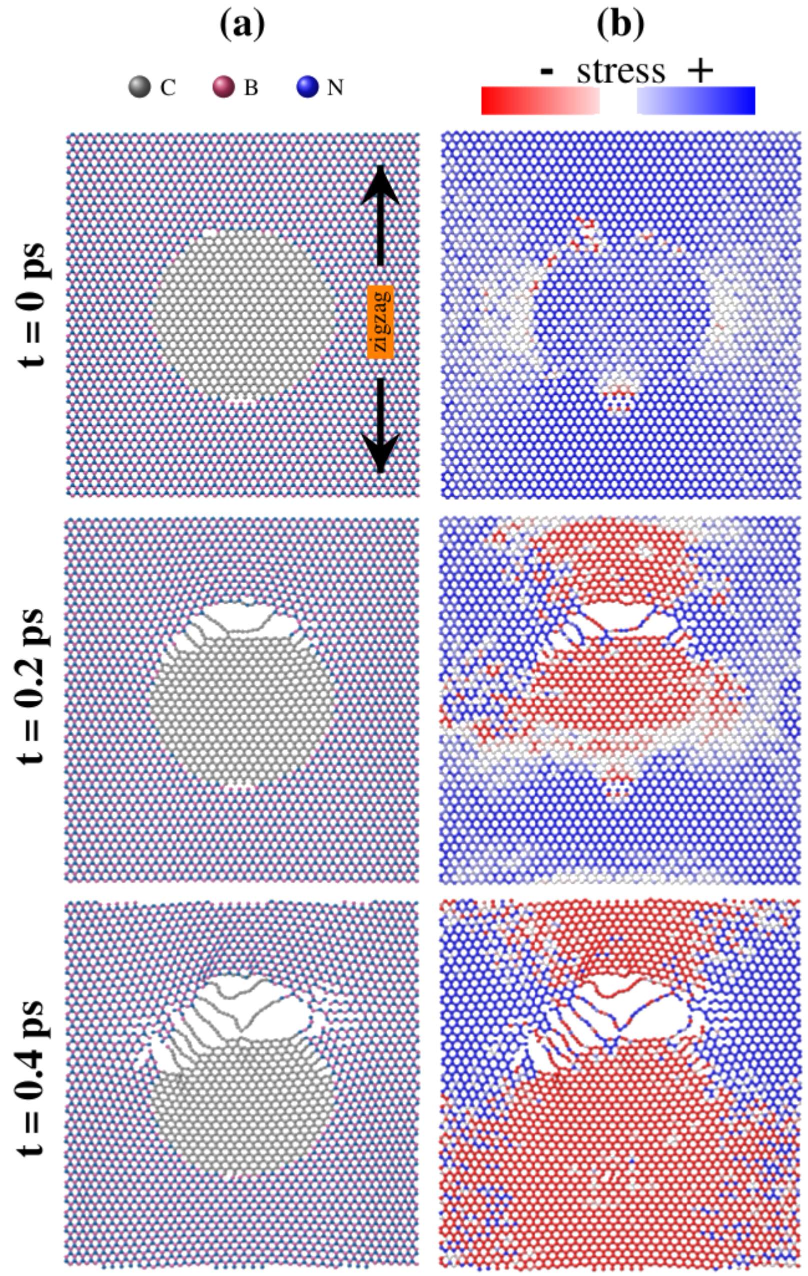

FIG. S4. Fracture results, for strain applied along the zigzag direction for the hybrid sheet with $L_x = L_y = 10$ nm and a circular graphene domain ($d = 5$ nm). (a) Snapshots from MD simulations detailing the time evolution of the monolayer fracture. (b) Corresponding stress distribution for the structures presented in (a).

* leonardo@fisica.ufrn.br

[1] C. Lee, X. Wei, J. W. Kysar and J. Hone, *Science*, 2008, **321**, 385.

[2] A. Falin, Q. Cai, E. J. G. Santos, D. Scullion, D. Qian, R. Zhang, Z. Yang, S. Huang, K. Watanabe, T. Taniguchi, et al., *Nature communications*, 2017, **8**, 15815.

[3] D. Akinwande *et al.*, *Extreme Mechanics Letters*, 2017, **13**, 42.
